# Monitoring of clinical efficacy and *in vitro *sensitivity of *Plasmodium vivax *to chloroquine in area along Thai Myanmar border during 2009-2010

**DOI:** 10.1186/1475-2875-10-44

**Published:** 2011-02-16

**Authors:** Poonuch Muhamad, Ronnatrai Ruengweerayut, Wanna Chacharoenkul, Kanchana Rungsihirunrat, Kesara Na-Bangchang

**Affiliations:** 1 Pharmacology and Toxicology Unit, Graduate Program in Biomedical Sciences, Thammasat University, Thailand; 2 Mae-Sot General Hospital, Mae-Sot, Tak Province, Thailand; 3 College of Public Health, Chulalongkorn University, Thailand

## Abstract

**Background:**

In Thailand, the proportion of *Plasmodium vivax *infection has become equal to *Plasmodium falciparum*. Reports of a trend of gradual decline of *in vitro *sensitivity of *P. vivax *to chloroquine in some areas of the country, together with accumulating evidences of chloroquine resistance *P. vivax *in other parts of the world, emphasize the need for closely and continuously monitoring clinical efficacy in conjunction with *in vitro *sensitivity of *P. vivax *isolates.

**Methods:**

The study was conducted at Mae Tao clinic for migrant workers, Tak Province during March 2008 - August 2009. A total of 130 patients (17 Thais and 113 Burmeses; 64 males and 66 females) with mono-infection of *P. vivax *malaria, aged between 15-60 years and weighing more than 40 kg, were included in the study. Patients received treatment with chloroquine (2,000 mg chloroquine phosphate over three days) and the anti-relapse drug primaquine (15 mg for 14 days). *In vitro *sensitivity of *P. vivax *isolates was evaluated by schizont maturation inhibition assay.

**Results:**

All patients showed satisfactory response to treatment. The cure rate was virtually 100% within the follow-up period of 42 days. Neither recurrence of *P. vivax *parasitaemia nor appearance of *P. falciparum *occurred during the investigation period. *In vitro *data showed a stable sensitivity of chloroquine in this area since 2006. Geometric mean and median (95% CI) values of IC_50 _for chloroquine were 100.1 and 134.7 (1.1-264.9) nM, respectively.

**Conclusion:**

*In vivo *results suggest that the standard regimen of chloroquine was still very effective for the treatment of blood infections with *P. vivax *in the Thai-Myanmar border area. *In vitro *sensitivity data however, raise the possibility of potential advent of resistance in the future. Regular monitoring of the chloroquine sensitivity of *P. vivax *is essential to facilitate the early recognition of treatment failures and to expedite the formulation of appropriate changes to the drug policy.

## Background

*Plasmodium vivax *is responsible for approximately 70 to 80 million cases of malaria worldwide annually, and is the major cause of human malaria in parts of Pacific region and Central and South America [1]. The disease is rarely life-threatening, but morbidity from prolonged illness and the possibility of relapses from a persistent hepatic form (hypnozoite) are of major concern. *Plasmodium vivax *can only infect reticulocytes, which limits parasitaemia, usually to densities lower than 100,000/ml blood. The relapses can occurs weeks, months or years after initial exposure [2]. In Thailand, the proportion of *P. vivax *infection has now been increasing and has become equal to *Plasmodium falciparum *since 1998. On the western border of Thailand, the incidence of *P. vivax *has recently been reported as 20 *per *1,000 population *per *year, similarly to that of *P. falciparum *[3]. The blood schizontocide chloroquine and tissue schizontocide primaquine have remained the mainstay chemotherapeutics for the treatment of *P. vivax *infection in Thailand for more than 60 years, with conserved clinical efficacy of virtually 100% [3-5]. To date, there has been no clinical-parasitological evidence of chloroquine-resistant *P. vivax *in Thailand. Nevertheless, a trend of gradual decline of *in vitro *sensitivity to chloroquine has been documented in some areas of the country, particularly Thai-Myanmar border [6,7]. Furthermore, the accumulating reports of chloroquine resistance *P. vivax *in other parts of the world during the past three decades including Papua New Guinea [8-11], Indonesia [12], Irian Jaya [13-16], Guyana South America [17], Peru [18], Colombia [19], India [20], Myanmar [21-23], Vietnam [24], Turkey [25], and Ethiopia [26] emphasize the need for closely and continuously monitoring clinical efficacy in conjunction with *in vitro *sensitivity of *P. vivax *isolates.

The objectives of the present study were to assess *in vivo *efficacy of first-line regimen of chloroquine given with primaquine, and *in vitro *susceptibility of *P. vivax *isolates in areas along the Thai-Myanmar border to chloroquine and the new antifolate WR99120.

## Methods

### Study site

The study was conducted at Mae Tao clinic for migrant workers, Tak Province during March 2008 - August 2009. Malaria is a serious imported medical problem in this area with a low and stable disease transmission with two seasonal peaks and forest-related during May-August and November-January of each year. *Anopheles minimus *and *Anopheles dirus *are the principal vectors. *P. falciparum *and *P. vivax *are the two predominant species with an incidence of 1:1, *Plasmodium malariae *is occasionally found and *Plasmodium ovale *is rare [3]. All age groups are affected and nearly all the *P. vivax *infections are symptomatic. The study was approved by the Ethics Committee of the Ministry of Public Health of Thailand.

### Assessment of *in vivo *efficacy of chloroquine/primaquine

#### Patient recruitment and follow-up

A total of 130 patients (17 Thais and 113 Burmeses; 64 males and 66 females) with mono-infection with *P. vivax *malaria, aged between 15-60 years (target population) and weighing more than 40 kg were included in the study. Inclusion criteria included a parasitaemia of 1,000-100,000 parasites/μl blood, no signs of severe disease, no anti-malarial treatment during the preceding four weeks, no history of hepatic or kidney diseases. Written informed consent for study participation was obtained from all patients. All were admitted to the clinic during the three-day course of chloroquine and were requested to return for follow-up on days 7, 14, 21, 28, 35 and 42 after treatment initiation. Patients who developed fever or signs/symptoms of malaria were asked to return to the clinic for malaria blood examination.

On enrolment, each patient underwent a physical examination. A symptom questionnaire was also completed. Parasite counts, body temperature and blood for *in vitro *sensitivity test (1 ml blood collected into sodium heparinized plastic tube).

#### Evaluation of clinical efficacy

The efficacy of chloroquine when given with primaquine for the treatment of *P. vivax *malaria were assessed by (i) the proportion of patients with cure (cure rate), *i.e*., those who showed clearance of parasitaemia in the peripheral blood and absence of reappearance of parasitaemia within 42 days after treatment initiation, (ii) the parasite clearance time (PCT: the time taken for the parasite count to fall below the level of microscopic detection), and (iii) the fever clearance time (FCT: the time taken for the temperature to return to normal, *i.e*., < 37.3°C and remain so for at least 24 hours).

### Treatment

Patients received treatment with chloroquine given concurrently with primaquine for eradication of hepatic stages of *P. vivax *parasites. Chloroquine (Government Pharmaceutical Organization of Thailand, 250 mg chloroquine phosphate *per *tablet) at a total dose of 2,000 mg given over 3 days (500 mg, four times at 0 and 6-12 hours on day-0, followed by 500 mg daily for two days) and primaquine (Government Pharmaceutical Organization of Thailand, 15 mg base given daily for 14 days starting from the second day of chloroquine treatment and then once daily until day-14). All drugs were administered with a glass of 250 ml drinking water under the supervision of medical staff. Subjects were closely observed for at least 30 minutes after each drug ingestion.

Patients whose parasitaemia was not cleared or those who had a reappearance of *P. vivax *were to be treated with the same regimen of chloroquine and primaquine but the dose of primaquine was to be increased to 20 mg. Patients who developed parasitaemia with *P. falciparum *during the investigation period were to be given a three-day treatment with oral artesunate (600 mg given in doses of 200 mg at 0, 6 and 24 hours) in combination with mefloquine (1,250 mg given as 750 and 500 mg at 6-8 hours apart).

### Laboratory investigations

Following the first dose of drug administration, parasite counts (from finger-prick blood samples) were performed in all patients at six hourly intervals until two consecutive slides yielded negative results, and then during the follow-up period until day 42. Blood films were stained with Giemsa and examined by light microscope. Asexual stages of *P. vivax *were counted against 1,000 erythrocytes in thin blood films or against 200 white blood cells in thick films. The examination was reported as negative only after at least 200 fields of the thick film had been examined without encountering a parasite. Parasite species, morphology, and parasitaemia were assessed by microscopic examination.

### *In vitro *drug sensitivity assay

The schizont maturation assay was performed with *P. vivax *field isolates collected from all patients using a modified method of Russell and colleagues [27]. The concentration of serum added to the culture medium was increased from 10 to 30%. *Plasmodium vivax *field isolates were tested for their sensitivities against chloroquine and WR99210 at the concentration ranges of 0-10,000 nM for chloroquine (chloroquine phosphate: Liverpool School of Tropical Medicine, University of Liverpool, UK) and 0-2,560 nM for WR99210 (Jacobus Pharmaceutical Inc, Princeton, NJ, USA). The dose-response curve was analysed by nonlinear regression analysis using CalcuSyn™software (Biosoft™, Cambridge, UK). The results were expressed as inhibitory concentrations (IC) 10, 50 and 90, which are defined as the concentrations of chloroquine or WR99120 producing 10, 50 and 90% inhibition of parasite development as compared to the control. Quality control of the assay was implemented by parallel determination of the IC_50 _of chloroquine against chloroquine-sensitive and chloroquine-resistant *P. falciparum *clones.

## Results

### Clinical efficacy

A total of 130 patients with *P. vivax *malaria were included in the study and all had completed a 42 days follow-up period. Demographic and clinical data are summarized in Table 1. All patients showed good response following treatment with median (95% CI) values for FCT and PCT of 30 (18-36) and 24 (12-40) h, respectively. The cure rate following treatment was 100%; there was no patient with reappearance of parasitaemia during the 42 days follow-up. None developed *P. falciparum *parasitaemia during the investigation period.

**Table 1:** Demographic and clinical data of patients with *P. vivax *infection included in the study.

	Number (%) or median (95%CI)
***Patient characteristics:***	

Number included	130 (64 females, 66 males) (17 Thais, 113 Burmeses)

Age [years: median (95% CI)]	22 (15-55)

Admission parasitaemia [/μl: median (95% CI)]	4,898 (1,206-29,480)

***Clinical outcome:***	

Number (%) completed 42 days follow-up	130 (100)

Number (%) cured by day 42	130 (100)

PCT [h: median (95% CI)]	30 (18-36)

FCT [h: median (95% CI)]	24 (12-42)

Data are presented as number (%) or median (95% CI).

PCT (parasite clearance time): The time taken for the parasite count to fall below the level of microscopic detection

FCT (fever clearance time): the time taken for the temperature to return to normal, i.e., <37.3°C and remain so for at least 24 hours

### *In vitro *sensitivity

*In vitro *sensitivity assay was performed in all of the 130 fresh *P. vivax *isolates collected; 32 (24.6%) with initial parasitaemia between 2,120 and 64,360 parasites/μl were successfully assessed for *in vitro *sensitivity to chloroquine and WR99120. All isolates completely developed to mature schizont within 20-40 h depending on dominant stage prior to testing. Despite the variation of the started parasite stages, abnormality in parasite morphology was observed in all wells exposed to the test drugs. Table 2 summarizes the IC_10_, IC_50 _and IC_90 _values of chloroquine and WR99120. Marked variation in IC_50 _values were observed for both chloroquine and WR99120. Geometric mean and median (95% CI) values of IC_50 _for chloroquine were 100.1 and 134.7 (1.1-264.9) nM, respectively. Based on the cut-off IC_50 _of 100 nM used to classify chloroquine resistance in *P. falciparum *[28], 20 and 12 isolates, respectively exhibited IC_50 _values of < and > 100 nM, respectively. Geometric mean and median (95%CI) values of IC_50 _for WR99120 were 112.7 and 139.9 (0.2-523.0) nM, respectively.

**Table 2:** Sensitivity of *P. vivax *to chloroquine and WR99120; data are presented as geometric mean and median (95%CI) values of IC_10_, IC_50 _and IC_90 _obtained from 32 isolates.

Drugs	Inhibitory Concentration (IC)	Geometric mean (nM)	Median (range) (nM)
Chloroquine	IC_10_	3.3	4.2 (0.2-7.1)
	
	IC_50_	100.1	134.7 (1.1-264.9)
	
	IC_90_	2956.5	4134.9 (7.1-11535.0)

WR99210	IC_10_	4.9	5.7 (0.04-17.8)
	
	IC_50_	112.7	139.9 (0.2-523.0)
	
	IC_90_	2585.0	3406.9 (1.1-15356.0)

IC _10_, _50 _and _90_: drug concentrations which produced 10, 50 and 90% inhibition of parasite development, respectively, as compared to the control (analysed by nonlinear regression analysis using CalcuSyn™software).

## Discussion

Difficulty in controlling vivax malaria is due to its biological characteristics of repeated pre-erythrocytic development due to relapse, with the maximum survival span of hypnozoites of five years [3]. Radical treatment of the infection, therefore, normally consists of a blood schizontocidal course of chloroquine and a course of primaquine for the elimination of the hypnozoites as antirelapse therapy [29]. In most parts of the world, chloroquine remains the first-line treatment for *P. vivax *infection in pregnant and non-pregnant individuals [1]. In the present study, all of the *P. vivax *patients enrolled in the study were satisfactorily treated with the standard regimen of chloroquine with primaquine. All responded well to treatment, with no reappearance of *P. vivax *parasitaemia (recrudescence or relapse) or appearance of *P. falciparum *in peripheral blood during the 42 days follow-up. There was no sign of delayed parasitological and clinical response. The PCT (30 h) and FCT (24 h) were similar to our previous observation in 2006 [5] and a recent observation [30] in the same area. Double infection with *P. falciparum *and *P. vivax *is common in certain malaria-endemic areas of Thailand. The occurrence of subsequent *P. falciparum *following treatment of *P. vivax *malaria has been reported to be less frequent than that of *P. vivax *after treatment of *P. falciparum *[31]. One possible explanation is the action of primaquine on pre-erythrocytic and eryhtrocytic forms of *P. falciparum *[32]. In addition, all patients stayed in malaria non-endemic area during the follow up period, which excluded the possibility of re-infection. So far, there has been no reported case of chloroquine resistant *P. vivax *in Thailand. Data from previous studies in different endemic areas including Thai-Cambodian and Thai-Myanmar borders during 1989 to 2011 [4-7,32] all indicated full sensitivity of *P. vivax *to standard dose of chloroquine. In a recent multicenter randomized, double-blind, non-inferiority trial conducted in Cambodia, India, Indonesia and Thailand (Mae Sot and Mae Ramat), a 42-day cure rate of chloroquine given with primaquine was 100% [32]. Nevertheless, a recent clinical trial in Ethiopia confirmed resistance of *P. vivax *to chloroquine with a 28-day cure rate of 7.5% [33]. Reappearance of *P. vivax *parasitaemia beyond day 28 which is suggested to be due to true relapse due to primaquine failure [29], and the relapse rates within 1-6 months were reported to be 5-18% in adult patients both in Thailand and in other tropical areas [14,29]. Clinical effectiveness of primaquine as an anti-relapse and patients' compliance could not be evaluated in this short follow-up investigation period.

The *in vitro *sensitivity data based on schizont maturation inhibition test [27] demonstrated more or less the stability of sensitivity of *P. vivax *isolates in this area of Thailand to chloroquine [geometric mean IC_50 _of 100.1, median (95%CI) of 134.7 (1.17-264.9) nM] since 2002 [27,34]. Sensitivity of *P. vivax *to chloroquine was shown to be increased by about 2-fold (IC_50_: from 131 to 71 nM) in the presence of primaquine, which also possesses direct blood schizontocidal activity [5]. It was noted that the IC_50 _of chloroquine in *P. vivax *was about 2-fold of that of *P. falciparum*, but the variation is probably higher with *P. vivax *[35]. A previous study conducted in the same area for monitoring of *in vitro *susceptibilities and molecular markers of resistance of *P. falicparum *isolates to chloroquine, quinine, mefloquine and artesunate [35] showed the reversed sensitivity of chloroquine after a period of about 40 years withdrawal from first-line treatment for falciparum malaria, with median (95% CI) IC_50 _of chloroquine of 73 (10-164) nM. One (4%), 19 (73%) and 6 (23%) isolates were classified as chloroquine-sensitive, moderately resistant and highly resistant *P. falciparum*, respectively. In other studies, about 3-4 fold higher IC_50 _of chloroquine was observed in *P. vivax *compared with *P. falciparum *[35,36]. This may imply intrinsic characteristic (innate resistance) of *P. vivax *in response to chloroquine. The *in vitro *cut-off value defining clinically relevant chloroquine resistance in *P. vivax *malaria has yet to be clearly defined. For *P. falciparum*, cut-off IC_50 _of 100 nM was used to define chloroquine resistance. Suwanarusk and colleagues [37] defined the cut-off IC_50 _of 220 nM based on the 35^th ^percentile of the clinical failure rate of 65% observed in Indonesian patients with *P. vivax *malaria. In the same study [37], the IC_50 _of chloroquine in Thai isolates collected from Mae Sot District, Thai-Myanmar border (same area as the present study) was also found to be significantly lower than that from Indonesian isolates (geometric mean IC_50 _of 312 *vs *46.8 nM). Eleven out of 81 Thai isolates (13.6%) exhibited IC_50 _of chloroquine over 220 nM. Based on this criteria, six out of 32 isolates (18.8%) observed in the present study showed IC_50 _of greater than 220 nM. The current *in vivo *and *in vitro *results suggest that chloroquine is still an effective first-line treatment for *P. vivax *in Thailand. Resistance level may remain obviously below the threshold of detectability by the *in vivo *method. It is noted that definitive conclusion on the efficacy of chloroquine is not appropriate since chloroquine was given with primaquine, and the fact that the study design did not include control arm with chloroquine monotherapy due to ethical reason. Regular monitoring of the chloroquine sensitivity of *P. vivax *is essential as to facilitate the early recognition of treatment failures and to expedite the formulation of appropriate changes to the drug policy. Alternative treatment options for *P. vivax *infection in case of chloroquine resistance may include a three-day course of quinine given concurrently with primaquine [5] or artemisinin combination therapy [38].

Various *in vitro *assay systems with different endpoint criteria have also been applied for monitoring of sensitivity of *P. vivax *isolates to anti-malarial drugs. Direct comparison of *in vitro *sensitivity data using different methods should however, be interpreted with caution. Since *P. vivax *infection is predominantly asynchronous, the microscopic method based on inhibition of parasite's growth previously developed by our group [39] is considered the best-suited method for assessing sensitivity of *P. vivax *to anti-malarial drugs [40]. The test method based on schizont maturation inhibition used in the present study, although may be less accurate, but the method is extremely less labour-intensive, more applicable for field studies and does not require expensive or dangerous reagents (monoclonal antibodies or radioisotopes). Unluckily, the success rate of *in vitro *sensitivity test observed in the current study was relatively low (24.6%), which is possibly due to variation in asynchronicity of parasite isolates in this area. Russel *et al *[41] demonstrated the marked stage-specific activity of chloroquine with variable growth rates. Isolates initially at the trophozoite stage had significantly higher chloroquine IC_50 _values than those initially at the ring stage. Synchronous isolates which reached the target of 40% schizonts in the control wells within 30 hours had significantly higher geometic mean IC_50 _of chloroquine. *In vitro *susceptibility was found to be correlated with initial stage of the parasite, with isolates predominantly at the trophozoite stage having a 2-fold increase in IC_50 _values compared to those of parasites predominantly at the ring stage [41].

The spread of chloroquine resistance in *P. falciparum *has led to the use of the antifolate combination sulphadoxine-pyrimethamine (SP) as the first-line drug for malaria treatment in several countries including Thailand, where *P. vivax *and *P. falciparum *often co-exist and occur at approximately equal frequencies [3]. *In vitro *sensitivity of WR99210, a novel inhibitor of enzyme dihydrofolate reductase (DHFR) was assessed in our study in view of previous reports showing its promise as a possible treatment of *P. vivax *malaria. The drug shows activity against the most pyrimethamine-resistant *P. falciparum *strains and extremely effective inhibitor of the *P. vivax *DHFR including mutations that confer high-level resistance to pyrimethamine [42,43]. Median (95% CI) IC_50 _of WR99120 in *P. vivax *isolates collected in the present study was 139.9 (0.21-523.0) nM. The relatively poor *in vitro *susceptibility of *P. vivax *to WR99120 observed was similar to our previous observation in the same area [44], could be explained by the slow action of this drug and/or the innate resistance as well as the presence of *p*-aminobenzoic acid and folate in the media used which acted as competitive antagonists of antifolate activity [45].

The combination of new antifolates, like WR99210, that are effective against SP-resistant parasites, with appropriate partners, may also play an important role in a rational drug treatment strategy.

## Conclusions

Chloroquine is still sufficiently effective for blood schizontocidal therapy in areas along the Thai-Myanmar border. *In vitro *sensitivity data however raise the possibility of potential advent of resistance in the future. Regular monitoring of chloroquine sensitivity of *P. vivax *is essential.

## Competing interests

The authors declare that they have no competing interests.

## Authors' contributions

PM performed all the laboratory analysis. RR participated in patients' recruitment and sample collection. WC performed data analysis. KR participated in *in vitro *sensitivity analysis. KN participated in the design of the study, manage the study and finalize the manuscript. All authors read and approved the final manuscript.
